# A Small Molecule Coordinates Symbiotic Behaviors in a Host Organ

**DOI:** 10.1128/mBio.03637-20

**Published:** 2021-03-09

**Authors:** Katherine E. Zink, Denise A. Ludvik, Phillip R. Lazzara, Terry W. Moore, Mark J. Mandel, Laura M. Sanchez

**Affiliations:** a Department of Pharmaceutical Sciences, University of Illinois at Chicago, Chicago, Illinois, USA; b Department of Chemistry & Biochemistry, University of California, Santa Cruz, California, USA; c Department of Medical Microbiology and Immunology, University of Wisconsin-Madison, Madison, Wisconsin, USA; d University of Illinois Cancer Center, University of Illinois at Chicago, Chicago, Illinois, USA; e Northwestern University Feinberg School of Medicine, Chicago, Illinois, USA; Baylor College of Medicine

**Keywords:** mass spectrometry, specialized metabolites, symbiosis

## Abstract

The lifelong relationship between the Hawaiian bobtail squid Euprymna scolopes and its microbial symbiont Vibrio fischeri represents a simplified model system for studying microbiome establishment and maintenance. The bacteria colonize a dedicated symbiotic light organ in the squid, from which bacterial luminescence camouflages the host in a process termed counterillumination. The squid host hatches without its symbionts, which must be acquired from the ocean amidst a diversity of nonbeneficial bacteria, such that precise molecular communication is required for initiation of the specific relationship. Therefore it is likely there are specialized metabolites used in the light organ microenvironment to modulate these processes. To identify small molecules that may influence the establishment of this symbiosis, we used imaging mass spectrometry to analyze metabolite production in V. fischeri with altered biofilm production, which correlates directly to colonization capability in its host. “Biofilm-up” and “biofilm-down” mutants were compared to a wild-type strain, and ions that were more abundantly produced by the biofilm-up mutant were detected. Using a combination of structural elucidation and synthetic chemistry, one such signal was determined to be a diketopiperazine, cyclo(d-histidyl-l-proline). This diketopiperazine modulated luminescence in V. fischeri and, using imaging mass spectrometry, was directly detected in the light organ of the colonized host. This work highlights the continued need for untargeted discovery efforts in host-microbe interactions and showcases the benefits of the squid-*Vibrio* system for identification and characterization of small molecules that modulate microbiome behaviors.

## Abstract (precis)

The complexity of animal microbiomes presents challenges to defining signaling molecules within the microbial consortium and between the microbes and the host. By focusing on the binary symbiosis between Vibrio fischeri and *Euprymna scolopes*, we have combined genetic analysis with direct imaging to define and study small molecules in the intact symbiosis.

## INTRODUCTION

Nearly all animals are hosts to microbial species (1). Communities of microbes can inhabit host organs intracellularly or extracellularly, may confer benefits to the host, and collectively are referred to as the microbiome (2). Some of these relationships have been explored by investigating the role that chemical communication plays between species for ascertaining whether to initiate interactions or to maintain them (3). For instance, the plant pathogen Ralstonia solanacearum produces a hybrid nonribosomal peptide synthetase-polyketide synthase (NRPS-PKS) lipopeptide that induces chlamydospore development in soil-dwelling filamentous fungi, which may help it persist in the soil environment. Conversely, the fungal species Fusarium fujikuroi and Botrytis cinerea produce bikaverin to inhibit invasion of ralsolamycin-producing strains of R. solanacearum (4, 5). In cheese rind-derived microbial interactions, small molecules like zinc-coproporphyrin III and other siderophores mediate trace metal acquisition between bacteria and fungi (6, 7). In both of these cases, specialized metabolites were central to understanding the observed phenotypes between the domains of life. Beyond antagonistic relationships, in many bacterial species, the density of the population is monitored by self-secreted “autoinducer” compounds, which include homoserine lactones in Gram-negative bacteria and small peptides in many Gram-positive organisms (8). Quorum sensing was discovered in Vibrio fischeri, a marine Gram-negative bacterium that colonizes squid and fish hosts (9). Studying V. fischeri in the context of its natural hosts provides an opportunity to expand our knowledge of biologically relevant compounds that influence microbial behaviors and microbe-host signaling in animal microbiomes.

To study the role of specialized metabolites in microbiome-host interactions, we have focused on the binary partnership between V. fischeri and the Hawaiian bobtail squid, *Euprymna scolopes*. Shortly after hatching, the squid host acquires the symbiont from the seawater, and the microbe proceeds to colonize the epithelial-lined crypts of the light organ. The bacteria gain a safe nutrient-rich habitat in which they can replicate (10). In turn, the bacteria in the aptly named “light organ” produce luminescence for the squid. The bacterial light camouflages the nocturnal predator via counterillumination, hiding the animal’s shadow in the moonlight so that it is less visible to predators and prey while the squid forages (11). Many aspects of the colonization process are comparable to bacterial colonization of human epithelial cells in the gut and on skin (3, 12, 13). As a naturally coevolved relationship where one host organ houses only one bacterial species, the simplified *Vibrio*-squid system allows for analysis of specialized metabolites involved in colonization of epithelial tissues. The colonization process is highly efficient and the host tissues are largely transparent, facilitating imaging approaches during the early stages of colonization. V. fischeri is amenable to genetic manipulation, including mimicking of symbiotic behaviors in culture. Furthermore, both partners can be raised separately and then mixed in a controlled fashion, enabling well-controlled experiments.

There is remarkable colonization specificity in that only V. fischeri—and only specific strains of V. fischeri—colonize the *E. scolopes* light organ (14). Part of this specificity lies in the exchange of chemical cues between the two partners that leads to maturation of the symbiosis, including the release of bacterial products peptidoglycan, the peptidoglycan monomer tracheal cytotoxin (TCT), lipopolysaccharide (LPS), while the host releases nitric oxide (NO) and chitin (15–20). A major checkpoint for this specificity is that bacterial biofilm production is required for bacterial aggregation and subsequent colonization (21, 22). Bacterial aggregates were first observed during confocal imaging of V. fischeri strain ES114 colonization of squid (23), and the genetic basis for aggregate formation was shown to require the hybrid histidine kinase RscS, hybrid histidine kinase SypF, response regulator SypG, and the 18-gene target symbiosis polysaccharide (*syp*) locus (21, 24, 25). Strains lacking any of the above regulators are unable to colonize squid robustly. Recent work has identified additional biofilm regulators, including BinK and HahK, which interface with the above pathway. BinK is a strong negative regulator of biofilm formation; in strains lacking BinK, increased biofilm formation is observed, whereas overexpression of BinK leads to reduced or absent biofilm development (26). HahK mediates NO signaling from the squid host via the HnoX NO sensor; in the presence of NO, HnoX inhibits HahK’s activity to promote biofilm formation, thus leading to enhanced dispersal of the symbiotic biofilm (27). The work on NO highlights the importance of small signaling molecules in the biofilm at the host interface.

We hypothesized that additional small molecule production may contribute to the propensity for V. fischeri to outcompete other bacteria. An experimental advantage offered by the *Vibrio*-squid system is the ability to investigate symbiotic phenotypes in culture-based assays that closely mirror the behavior in the host. Characterization of the biological roles for the positive regulator RscS and the negative regulator BinK were facilitated by culture-based assays that reflected the symbiotic phenotypes. Specifically, when RscS was overexpressed (using either a plasmid-based or chromosomal rscS* allele), V. fischeri produced large aggregates and outcompeted wild type in the squid. These same strains form visible biofilms on agar that manifest as wrinkled colonies. At the other end of the spectrum, strains that are interrupted in the gene encoding the RscS positive regulator are unable to form biofilm in culture or in the animal. Therefore, by using a set of genetically altered V. fischeri ES114 derivatives, we were able to examine a spectrum of biofilm phenotypes in culture that are relevant for the symbiotic behavior in the host. The three isogenic derivatives that were the focus for discovery in this paper are as follows. (i) A strain that does not form symbiotic biofilm in culture or in the animal (“biofilm-down”; i.e., ES114 rscS* *rscS*::Tn*erm*). This strain contains an interruption in *rscS* that blocks biofilm signaling. (ii) A strain that forms symbiotic biofilm in the animal but not in culture (“wild type” [WT]; i.e., unaltered ES114). (iii) A strain that forms symbiotic biofilm in culture and forms enhanced aggregates in the animal due to overexpression of the positive regulator RscS and the lack of the negative regulator BinK (“biofilm-up”; i.e., ES114 rscS* Δ*binK*) (26). We have shown previously that a similar gradient of strains was useful for revealing biofilm phenotypes that are relevant *in vivo* (26), and here we integrated these defined strains with cutting edge analytical technologies for the discovery and characterization of small molecules connected to symbiotic behaviors.

One method that can rapidly determine differences in metabolite production of microbes is agar-based imaging mass spectrometry (IMS). This approach has been applied to decipher microbe-microbe chemical communication and changes in metabolite production between microbial colonies grown in different environmental conditions (6, 28, 29). IMS is especially valuable for the comparison of metabolites of defined strains because changes in production, as measured by ion intensity, can be specifically and rapidly detected and evaluated in the context of genetic differences (30). IMS analysis of WT V. fischeri compared to biofilm-up and biofilm-down strains revealed that several metabolites were significantly upregulated in the biofilm-up strain compared to the other strains. These metabolites were hypothesized to be important for the production of the symbiotic biofilm and/or the increased fitness of the biofilm-up mutant in the light organ environment. Additionally, because of the small size of *E. scolopes* hatchlings and the anatomical accessibility of the light organ, a whole-body imaging approach was optimized for *in vivo* investigation of the light organ (31). Together, these two IMS approaches were utilized to determine differences in specialized metabolism in V. fischeri mutants, both *in vitro* and *in vivo*, and led to the isolation and structural elucidation of a small molecule that increases V. fischeri luminescence, which may provide a fitness advantage in host-microbe recognition.

## RESULTS AND DISCUSSION

### Untargeted spatial metabolomics of V. fischeri biofilm mutants.

**FIG 1:**
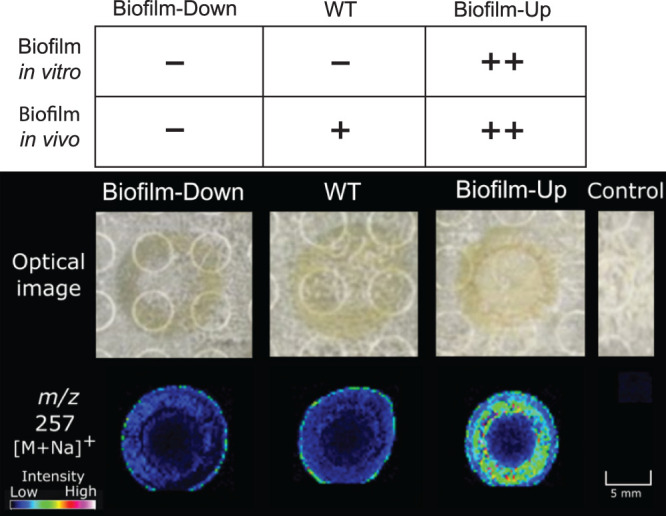
The *m/z* 257 from the mass panel in Fig. S1 was statistically more abundant in a biofilm-up strain compared to WT and biofilm-down strains (*n* = 4) (see the Materials and Methods for detailed genotypes). Using the “colocalization” function in SCiLS Lab (Bruker), the *P* value of *m/z* 257 in biofilm-up compared to WT and biofilm-down is 0.05 < *P* < 0.1, based on manual adjustment of the significance threshold.

IMS was employed to generate a rapid screen of mass-to-charge ratios (*m/z*) in WT, biofilm-up, and biofilm-down V. fischeri strains to identify ions that differ between solid agar colonies of the three strains. A positive mode analysis in the small molecule range (100 to 1,000 Da) generated a panel of masses that were significantly more abundant (*P* < 0.1) in the biofilm-up mutant than in the other two samples, as determined by the “colocalization” function in SCiLS software (Bruker). Figure S1 in the supplemental material displays seven of these significant features that replicated at least twice across four biological replicates. One of these signals, *m/z* 257, was detected with high signal intensity in biofilm-up, and low signal intensity in WT and biofilm-down (Fig. 1). We proceeded to validate this compound using a different model of biofilm induction. It was recently shown that calcium in the medium can stimulate V. fischeri biofilm formation in strains lacking BinK, but without the need for overexpression of rscS* (32). This both provided an independent biofilm model in which we could test for production of the compound, and additionally allowed us to ask whether the compound was produced by other natural isolates, given that the rscS* overexpression approach does not apply in all backgrounds (33). In ES114, we observed elevated abundance of the compound on the calcium medium upon biofilm induction (i.e., in the strain lacking BinK), supporting the use of this model (Fig. S2). We also detected elevated compound in biofilm-induced strains MB11B1, MB15A4, and SR5 (Fig. S2). Additionally, we detected the compound in strain ES213 but no increase was detected upon biofilm induction (Fig. S2). These results therefore report that this compound is reliably detected in multiple biofilm models and can be detected in strains representing the three major phylogenetic groups of symbiotic V. fischeri (33). This signal was therefore prioritized for dereplication, the process of identifying known unknowns, because biofilm production is strongly correlated with a colonization advantage.

10.1128/mBio.03637-20.1 FIG S1 Prioritized mass features detected using IMS. Download FIG S1, PDF file, 0.3 MB. Copyright © 2021 Zink et al. 2021 Zink et al. https://creativecommons.org/licenses/by/4.0/ This content is distributed under the terms of the Creative Commons Attribution 4.0 International license.

10.1128/mBio.03637-20.2 FIG S2 Analysis of natural V. fischeri isolates. Download FIG S2, PDF file, 0.4 MB. Copyright © 2021 Zink et al. 2021 Zink et al. https://creativecommons.org/licenses/by/4.0/ This content is distributed under the terms of the Creative Commons Attribution 4.0 International license.

### Dereplication and structure elucidation of cyclo(histidyl-proline).

Masses identified via IMS were prioritized to a select list by evaluating their statistical significance. Because ionization capacities change with different modalities (e.g., matrix-assisted laser desorption ionization [MALDI] versus electrospray ionization [ESI]) liquid chromatography-tandem mass spectrometry (LC-MS/MS) data of crude V. fischeri extracts from each mutant were queried using Global Natural Products Social molecular networking (GNPS) to investigate signals detected in IMS, as well as to probe compounds that may only ionize in ESI. A spectral match from the V. fischeri biofilm-up extract was detected for a small molecule, cyclo(histidyl-proline) (cHP), with a molecular weight of 234 g/mol (Fig. S3) (34, 35). The precursor ion from GNPS (*m/z* 235) matched one of the statistically significant masses from IMS analysis; often, ions detected in IMS are sodiated adducts ([M+Na]^+^) because of salt added to the medium to support growth of marine bacteria. Therefore, the *m/z* 257 from Fig. 1 was likely the sodiated adduct of cyclo(His-Pro), and the protonated molecule was *m/z* 235 (Fig. S4). Of interest to this context, cyclo(His-Pro) is a member of the diketopiperazine (DKP) structural class. DKPs are formed from the cyclization of two amino acids and are prevalent natural products that play a variety of roles in microbial relationships (36).

10.1128/mBio.03637-20.3 FIG S3 GNPS match of *m/z* 235 to cyclo(His-Pro). Download FIG S3, PDF file, 0.3 MB. Copyright © 2021 Zink et al. 2021 Zink et al. https://creativecommons.org/licenses/by/4.0/ This content is distributed under the terms of the Creative Commons Attribution 4.0 International license.

10.1128/mBio.03637-20.4 FIG S4 MALDI-TOF dried droplet spectrum of *m/z* 235 in biofilm-up extract. Download FIG S4, PDF file, 0.3 MB. Copyright © 2021 Zink et al. 2021 Zink et al. https://creativecommons.org/licenses/by/4.0/ This content is distributed under the terms of the Creative Commons Attribution 4.0 International license.

**FIG 2:**
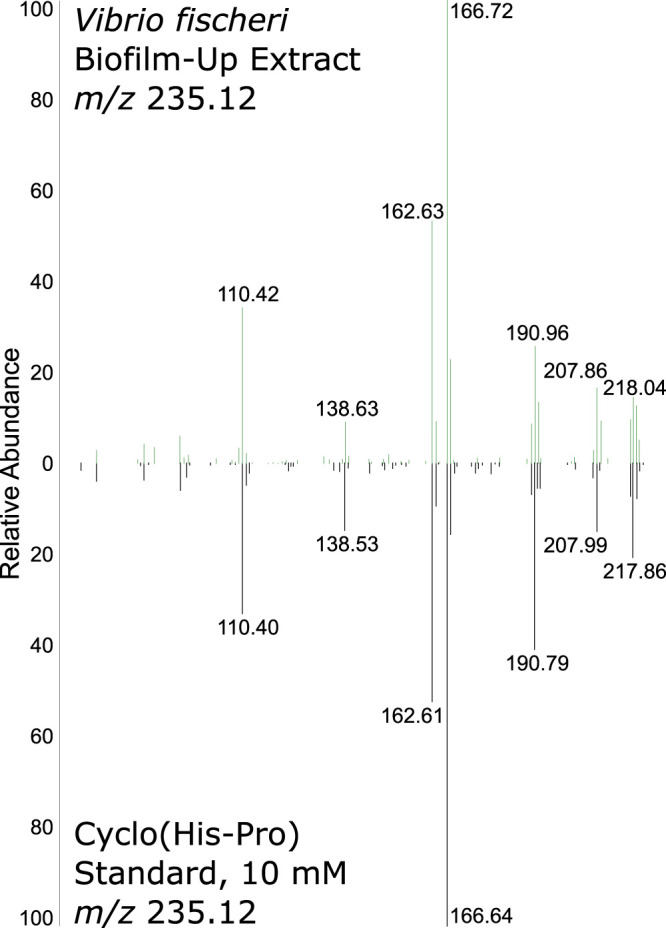
A standard of cyclo(His-Pro) mass fragmentation matched with the extracted molecule at *m/z* 235.12 using direct infusion.

While GNPS identified cyclo(His-Pro) as a putative assignment, we employed direct infusion to detect and confirm the fragmentation patterns of the *m/z* 235 from the V. fischeri biofilm-up extract and a commercial cyclo(l-His-l-Pro) standard. Figure 2 depicts a near identical match between the fragmentation patterns of both samples using direct infusion, which was validated using high-resolution electrospray ionization mass spectrometry (HRESIMS) (Fig. S5). The protonated precursor ion (*m/z* 235.12) and all high-intensity fragments matched between the standard and extracted samples. These data provided strong evidence to support a level 2 identification, as defined by the chemical analysis working group (CAWG), of cyclo(His-Pro) in the V. fischeri biofilm-up extract (37).

10.1128/mBio.03637-20.5 FIG S5 Extracted ion chromatograms of cyclo(His-Pro) on the Q-ToF mass spectrometer. Download FIG S5, PDF file, 0.4 MB. Copyright © 2021 Zink et al. 2021 Zink et al. https://creativecommons.org/licenses/by/4.0/ This content is distributed under the terms of the Creative Commons Attribution 4.0 International license.

**FIG 3:**
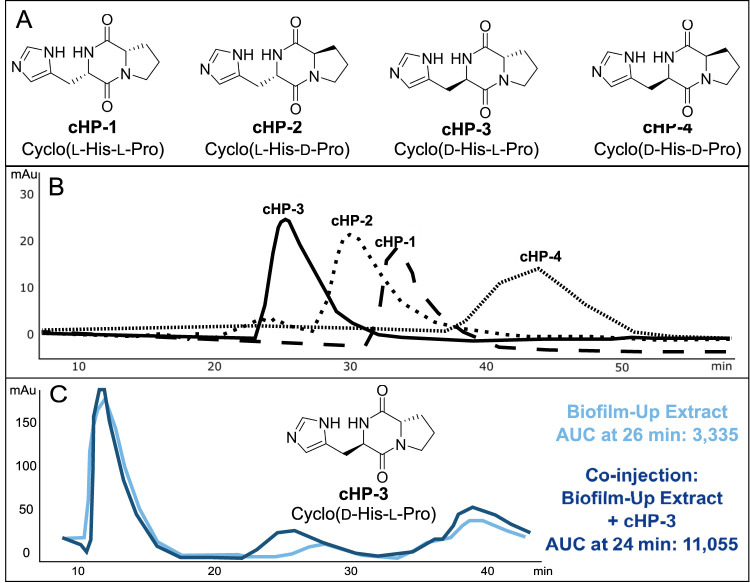
Retention time matching of V. fischeri biofilm-up extract on a chiral column indicates that the configuration of cyclo(His-Pro) in the microbial extract is stereoisomer cHP-3, cyclo(d-His-l-Pro). (A) Structures of all stereoisomers of cyclo(His-Pro). (B) Retention times of all synthesized stereoisomers: cHP-3 (24 min), cHP-2 (30 min), cHP-1 (33 min), and cHP-4 (44 min). A cellulose-B column was used to retain stereoisomers using 13:87 isopropyl alcohol (IPA):hexanes over 60 min at 2 ml/min. (C) A peak at 26 min was observed in the biofilm-up extract (light blue trace) and the area under the curve (AUC) measured 3,335. When coinjected with cHP-3 (dark blue trace), the peak at 26 min increased in AUC to 11,055, indicating the presence of cHP-3 in the biofilm-up extract. UV-vis was monitored at 214 nm.

To elucidate the stereochemical configuration of the DKP, as MS/MS is considered to be largely stereochemically blind, we synthesized all four possible stereoisomers of cyclo(His-Pro): (i) cyclo(l-His-l-Pro) (cHP-1); (ii) cyclo(l-His-d-Pro) (cHP-2); (iii) cyclo(d-His-l-Pro) (cHP-3); and cyclo(d-His-d-Pro) (cHP-4). The four stereoisomers were synthesized for chemical comparison to the isolated compound from the V. fischeri biofilm-up extract (Fig. 3A) (38). To validate the configuration of the synthetic material, each stereoisomer was analyzed using both nuclear magnetic resonance (NMR) and optical rotation (OR). Kukla et al. reported optical rotation values, which were used for comparison and validation of each isomer (Text S1) (38).

10.1128/mBio.03637-20.10 TEXT S1 General experimental, synthesis, and characterization of the different DKP stereoisomers. Download Text S1, PDF file, 1.0 MB. Copyright © 2021 Zink et al. 2021 Zink et al. https://creativecommons.org/licenses/by/4.0/ This content is distributed under the terms of the Creative Commons Attribution 4.0 International license.

Chiral chromatography was used to match the retention time of the DKP in the V. fischeri extract to all four synthesized stereoisomers (Fig. 3B). After comparison with each stereoisomer, it was determined that the peak of stereoisomer cHP-3 was also detected in the V. fischeri biofilm-up extract. When coinjected, the peaks from both samples coalesced to one peak, indicating that their structures and configuration were identical (Fig. 3C). This retention time matching provides a level 1 identification for the *m/z* 235 from the V. fischeri biofilm-up, stereoisomer cHP-3 (cyclo[d-His-l-Pro]). The other stereoisomers, namely cHP-2, were eliminated as being produced in the biofilm-up extract because either their retention time did not match an existing peak in the biofilm-up extract, or the coinjection peak did not coalesce with an extract peak (Fig. S6). Quantification of the molecule in extracts of the biofilms produced by the mutants also indicated that the biofilm-up mutant produced a larger amount of cHP (measured as m/z 235.12) (Fig. S7). To attempt to validate the role of cHP-3 in the *E. scolopes* host, we aimed to capture production of cHP-3 *in vivo*.

10.1128/mBio.03637-20.6 FIG S6 Retention time matching of cyclo(d-His-l-Pro) to biofilm-up. Download FIG S6, PDF file, 0.3 MB. Copyright © 2021 Zink et al. 2021 Zink et al. https://creativecommons.org/licenses/by/4.0/ This content is distributed under the terms of the Creative Commons Attribution 4.0 International license.

10.1128/mBio.03637-20.7 FIG S7 Quantification of cyclo(d-His-l-Pro) in V. fischeri wild type and mutants. Download FIG S7, PDF file, 0.3 MB. Copyright © 2021 Zink et al. 2021 Zink et al. https://creativecommons.org/licenses/by/4.0/ This content is distributed under the terms of the Creative Commons Attribution 4.0 International license.

### *In vivo* detection in hatchlings.

**FIG 4:**
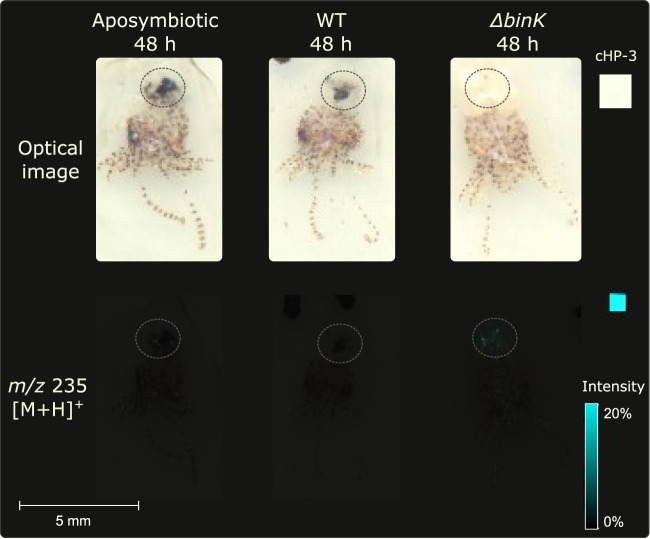
Representative replicates depicting *m/z* 235 (cHP-3) is detected *in vivo* in the light organ of *E. scolopes* hatchlings colonized both when inoculated with V. fischeri WT and (strongly) with WT Δ*binK* (*n* = 3). Dotted circles surround the light organ region in each hatchling. Colors in the light organ regions vary depending on level of ink removal. Replicates can be found in Fig. S8 in the supplemental material.

A recently developed sample preparation protocol for minimally manipulated whole-invertebrate-organism IMS was utilized to assess whether cHP-3 was being generated *in vivo* in the light organ of *E. scolopes* hatchlings (31). The presence of the ion in the light organ would indicate that cHP-3 has an ecological importance if it can be detected *in vivo*. Three conditions were evaluated: (i) no V. fischeri (aposymbiotic), (ii) V. fischeri WT, and (iii) V. fischeri WT Δ*binK* (analogous to the biofilm-up strain, but instead of genetically inducing biofilm formation with the rscS* allele, relies on induction from native signals in the squid host). *E. scolopes* hatchlings were inoculated with each sample for 3 h, washed, and allowed to continue to colonize for 48 h, as described previously (39). At 48 h, *m/z* 235 was not detected in the aposymbiotic control, was produced weakly in the WT condition, and was produced strongly in the WT Δ*binK* condition (Fig. 4). This was evidence that the molecule was produced by the WT strain *in vivo* and that the knockout of *binK* resulted in increased production of the ion.

10.1128/mBio.03637-20.8 FIG S8 Detection of cHP-3 in light organ replicates. Download FIG S8, PDF file, 0.3 MB. Copyright © 2021 Zink et al. 2021 Zink et al. https://creativecommons.org/licenses/by/4.0/ This content is distributed under the terms of the Creative Commons Attribution 4.0 International license.

The consistency in detection of cHP-3 production *in vitro* and *in vivo* is significant in light of the bacterial strains that were used. The WT strain is the same for both assays, whereas the comparative strain in each case lacks BinK. However, in the *in vitro* assay, biofilms are genetically induced with an rscS* allele (i.e., an overexpressed biofilm regulator), while this allele is not present for the strain used *in vivo* (Fig. 4). The rationale for this difference is that the activated RscS allele is used to mimic behavior in culture that is stimulated by the host conditions. Given that cHP-3 production is similarly induced in the host, we conclude that its production is a bona fide output of the symbiotic biofilm pathway and is induced in the host in both WT and in a Δ*binK* background, independent of artificial RscS activation.

### Role in stimulating bioluminescence.

Quorum sensing (QS) is the activity of bacterial cells engaging in group behavior facilitated by the production of QS molecules by individual cells until a concentration is reached to activate a particular pathway. In the case of V. fischeri, the LuxR pathway is activated and bioluminescent activity occurs. The most well studied QS molecules in the V. fischeri system are acyl-homoserine lactones (AHL), such as *N*-3-oxohexanoyl-l-homoserine lactone (OHHL). In other bacterial QS systems, proline-containing DKPs are responsible for activating the relative pathways. For example, cyclo(l-Pro-l-Leu) produced by Cronobacter sakazakii, cyclo(l-Pro-l-Tyr) and cyclo(l-Phe-l-Pro) produced by Pseudomonas aeruginosa, and cyclo(l-Pro-l-Val) isolated from *Haloterrigena halophilus* all influence the QS systems of other microbes (40–42). With so many structural combinations possible, there are many more examples of DKPs that influence microbial QS systems, some in biofilm formation, and some even in *Vibrio* spp. (43–46). For example, cyclo(l-Phe-l-Pro), which was originally isolated from V. vulnificus, was shown to activate the V. fischeri luminescence locus (47).

**FIG 5:**
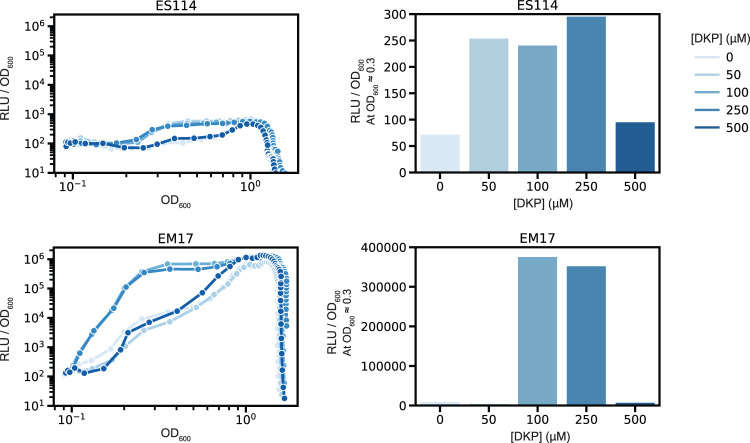
cHP-3 stimulates V. fischeri luminescence. Line graphs show cHP-3 increases relative light units in V. fischeri cultures at low concentrations in both a low-luminescence strain (ES114) and in a high-luminescence strain (EM17). Bar graphs show luminescence levels at a specific OD (OD_600_ = ∼0.3) to illustrate the concentration-dependent effect. Graphs are representative of three independent experiments.

Because of the relationship between biofilm production potential and appearance of cHP-3 in the colonized host, we sought to query whether cHP-3 affected the bioluminescence of V. fischeri. An increase in luminescence was observed at low cell densities in the WT strain (ES114) (Fig. 5). Since ES114 has low luminescence compared to other V. fischeri isolates, we tested the effect of cHP-3 in another strain, EM17, which is a much brighter isolate. This strain also exhibited an increase in luminescence with the addition of exogenous cHP-3 and the effect is much more pronounced than in ES114 (Fig. 5). The greatest effect on luminescence in both strains was seen with concentrations of 100 and 250 μM cHP-3. The effect of cHP-3 at low cell densities is similar for other quorum-sensing molecules, as there is a concentration threshold in these systems after which the addition of more compound does not increase activity (48). Given the optimal response at 250 μM for ES114, we asked whether exogenous addition of cHP-3 at this concentration would affect squid colonization. It did not impact colonization level or luminescence for the WT symbiont (Fig. S9). Given the high level of luminescence *in vivo*, we suspect that light production is saturated, and it will be necessary in future work to interrupt biosynthesis of cHP-3 to further investigate its role in the host.

10.1128/mBio.03637-20.9 FIG S9 Colonization of squid hatchlings with exogenous cHP-3. Download FIG S9, PDF file, 0.3 MB. Copyright © 2021 Zink et al. 2021 Zink et al. https://creativecommons.org/licenses/by/4.0/ This content is distributed under the terms of the Creative Commons Attribution 4.0 International license.

### Roles of microbial diketopiperazines.

Microbial DKPs have been detailed in a number of biological contexts, including production in sexual pheromone signaling, as enzyme inhibitors, and in quorum sensing (46, 49–51). Their structures vary widely, as there are many possible amino acid combinations and side chain modifications (36). A thorough understanding of the ecology of the squid-*Vibrio* system provides context for the potential role of cHP-3. With an observable increase in production of cHP-3 in a biofilm-up mutant, it is possible to surmise that cHP-3 may be critical for biofilm development, either as an end goal or as a preliminary step for a downstream process, or even as a quorum-sensing molecule. Bacterial DKPs have been implicated in a variety of roles. As an example, cyclo(l-Phe-l-Pro) was isolated from Vibrio vulnificus due to its ability to activate a V. fischeri luminescence locus (47). The compound is produced widely among *Vibrio* species, can affect production of toxin genes, and can influence host innate immunity (52–54). Several bacterially derived DKPs have been described as chitinase inhibitors: cyclo(l-Arg-l-Pro), cyclo(Gly-l-Pro), cyclo(l-His-l-Pro) (cHP-1), and cyclo(l-Tyr-l-Pro) (51, 55, 56). While we have not explored the chitinase inhibition activity of cHP-3, if the stereoselectivity is not important in other activities of DKPs, it is possible that cHP-3 displays the same inhibitory activity in the squid-*Vibrio* system, especially if cHP-1 is known to be inhibitory. Because *E. scolopes* generates chitin as a source of nutrients for V. fischeri, presumably inhibition of chitinase by the bacterium would result in fewer monomers for consumption which would be thought to decrease fitness. Although this hypothesis has not yet been tested, there is precedent for the regulation of chitin and chitinase activity in this system (57).

In conclusion, application of MALDI-IMS across a gradient of V. fischeri ES114 biofilm-producing derivatives identified a small molecule, cHP-3, that is produced in significant quantities in the biofilm-up strain. cHP-3 is a member of the DKP molecular class, members of which are increasingly ubiquitous across microbial species and have various and dedicated activities, including signaling, quorum sensing, and enzyme inhibition. The effect we observed on bacterial luminescence suggests that this DKP may link the early developmental process of biofilm formation with the later light production.

The biosynthetic origin of cHP-3 is an immediate focus for our future work. Oftentimes, DKPs are shunt products to larger nonribosomal peptide synthetase (NRPS)-derived molecules; however, in our case, no larger peptidic masses (based on MS/MS) have been detected in the culture broth as of yet, which makes this biosynthetic route unlikely. Future studies will also focus on the protein target for luminescence activity, as well as the role in the light organ. The investigation of differentially produced small molecules in this controlled, two-partner system provides a platform for an increased understanding of the mechanisms that may be responsible for animal-microbe partners to recognize one another and to maintain a specific, lifelong relationship.

## MATERIALS AND METHODS

### V. fischeri strains.

All strains for the initial screen and squid colonizations are derivatives of the MJM1100 isolate of V. fischeri ES114, an *E. scolopes* light organ isolate (58, 59). MJM1776 is the “biofilm-down” isolate, with genotype MJM1100 rscS* rscS::Tn*erm*. It was isolated as a mariner transposon insertion from pMarVF1 in the *rscS* gene in strain MJM1198 (Mattias Gyllborg and M.J.M., unpublished) (60). MJM2255 is the “biofilm-up” isolate with genotype MJM1100 rscS* Δ*binK*, which was described previously (26). MJM2251 is the Δ*binK* strain used in Fig. 4, with genotype MJM1100 Δ*binK* (26).

For the broader natural isolate assessment, wild type and Δ*binK* derivatives are listed, respectively, for each strain: ES114 (MJM1100, MJM2251 [26]); MB11B1 (MJM1130 [22], MJM3084 [33]); MB15A4 (MJM2114 [33], MJM3087); ES213 (MJM1117 [22], MJM3551); and SR5 (MJM1125 [22], MJM4037). Strains MJM3087 and MJM3551 were constructed by allelic exchange, with the resulting strains bearing an unmarked Δ*binK* allele. Strain MJM4037 is the Δ*binK*::*bar* derivative of MJM3751, which carries the Δ*binK*::*erm-bar* allele constructed via *tfoX*-based transformation (33, 61).

### Bioluminescence assays.

Two Vibrio fischeri strains, ES114 (WT) and Euprymna morsei symbiont EM17, were grown overnight in LBS at 25°C. Cultures were diluted 1/1,000 in seawater tryptone with adjusted osmolarity (SWTO) with various concentrations of cHP-3 from 0 to 500 μM (62). Samples were then transferred in triplicate to a Nunc clear bottom plate and measurements were taken by Biotek Synergy Neo2 plate reader. Relative luminescence (RLU) and optical density at 600 nm (OD_600_) were measured every 30 min for 22 h. Triplicates were averaged and the specific luminescence (RLU/OD_600_) was plotted as a function of the OD_600_.

### Data availability.

Data files for all IMS and LS-MS/MS analyses can be found in the MassIVE database under ID MSV000085327.
