# Supplementary material for: A Small Molecule Coordinates Symbiotic Behaviors in a Host Organ
Source: mBio. 2021 Mar 9;12(2):e03637-20. doi: 10.1128/mBio.03637-20 (PMC8092321; doi:10.1128/mBio.03637-20)
Supplement: FIG S1 [file mBio.03637-20-sf001.pdf]

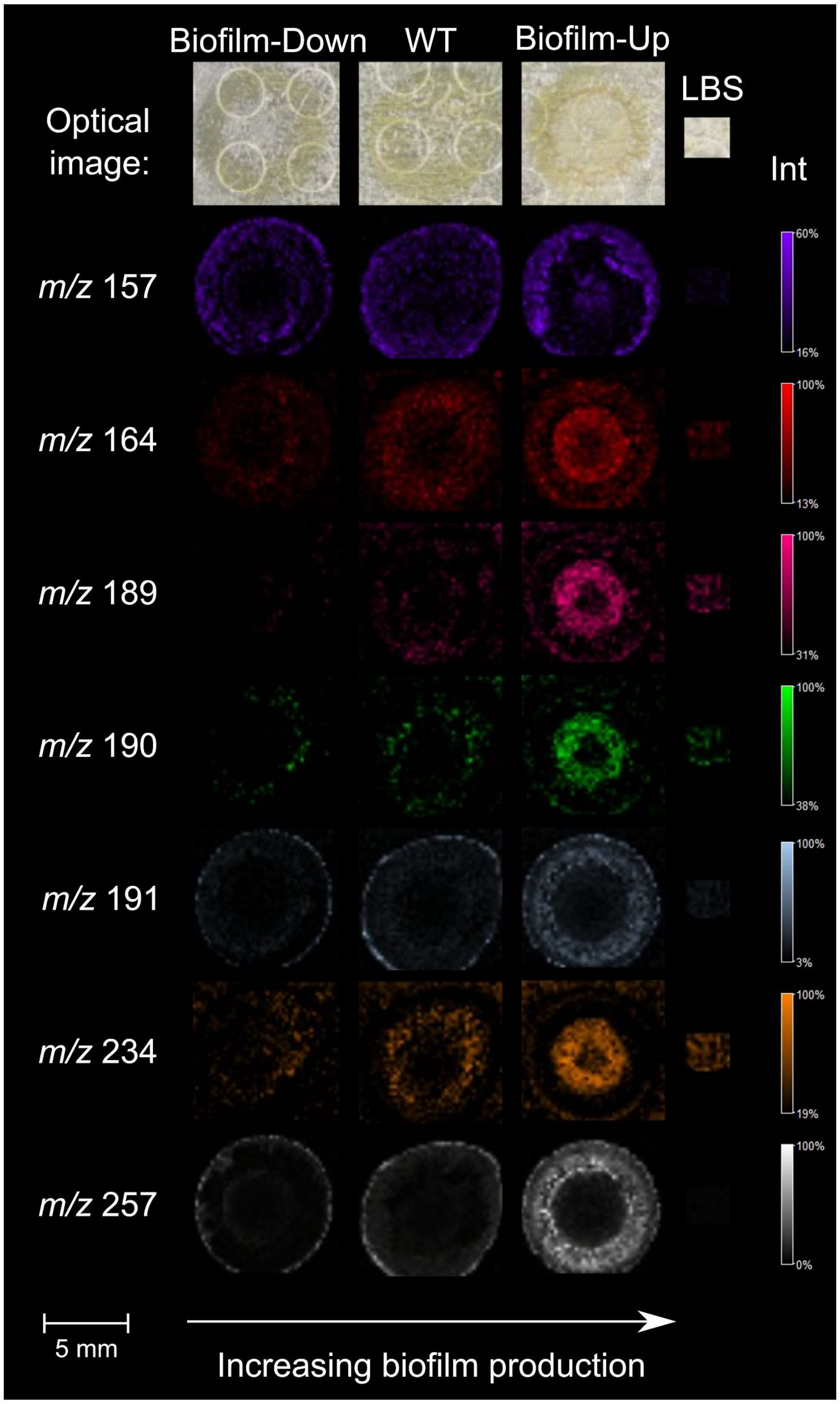

Figure S1. Seven small molecules were significantly more abundant ($p < 0.1$) in Biofilm-Up compared to WT and Biofilm-Down, detected as significant at least two across four biological replicates. IMS analysis was performed in positive mode and in the mass range of 100-1000 Da.
