# Supplementary material for: A Small Molecule Coordinates Symbiotic Behaviors in a Host Organ
Source: mBio. 2021 Mar 9;12(2):e03637-20. doi: 10.1128/mBio.03637-20 (PMC8092321; doi:10.1128/mBio.03637-20)
Supplement: FIG S2 [file mBio.03637-20-sf002.pdf]

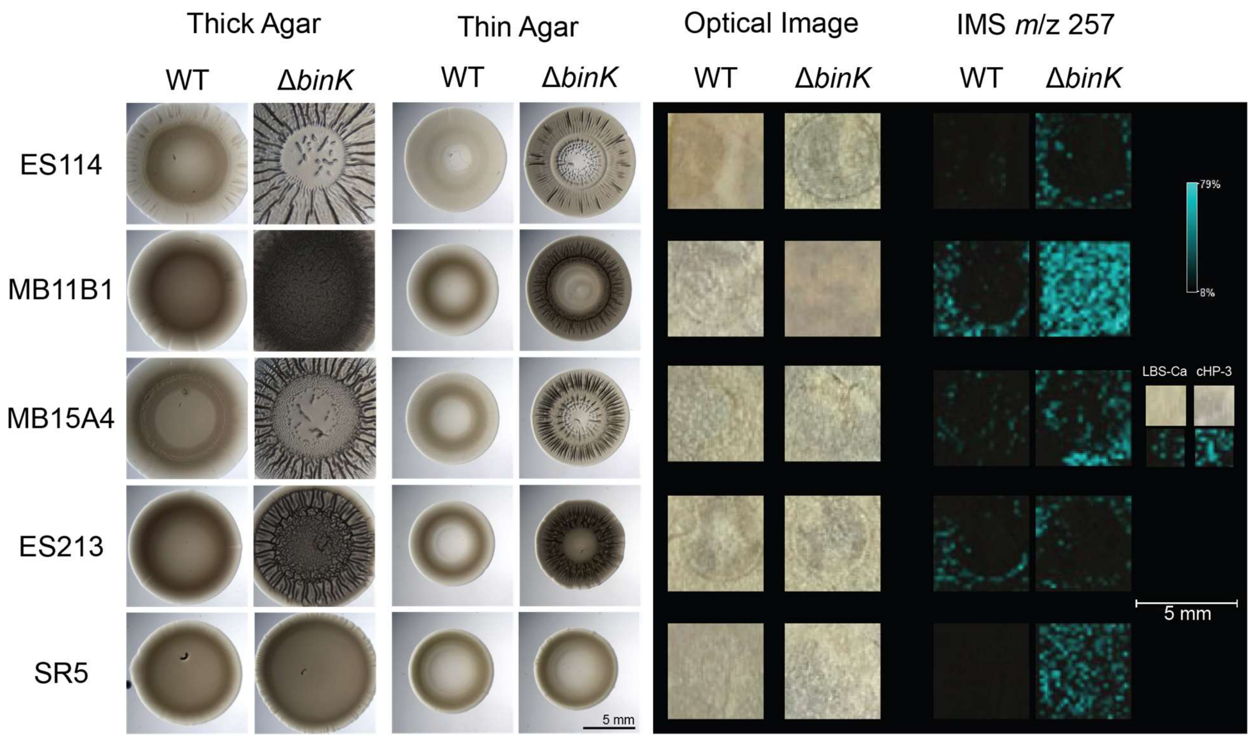

Figure S2. Detection of *m/z* 257 in diverse V. fischeri isolates. Biofilm was induced by addition of 10 mM calcium to the medium and deletion of *binK*. Wrinkled colony formation is evident on thick agar for most strains, and these same phenotypes can be replicated on the thin agar needed for IMS analysis. The *m/z* 257 compound is seen to be more abundant in strains lacking *binK* for all isolates except ES213. Colony images are representatives from multiple replicates grown at 25 °C and imaged at 96 h.
