# Supplementary material for: A Small Molecule Coordinates Symbiotic Behaviors in a Host Organ
Source: mBio. 2021 Mar 9;12(2):e03637-20. doi: 10.1128/mBio.03637-20 (PMC8092321; doi:10.1128/mBio.03637-20)
Supplement: FIG S3 [file mBio.03637-20-sf003.pdf]

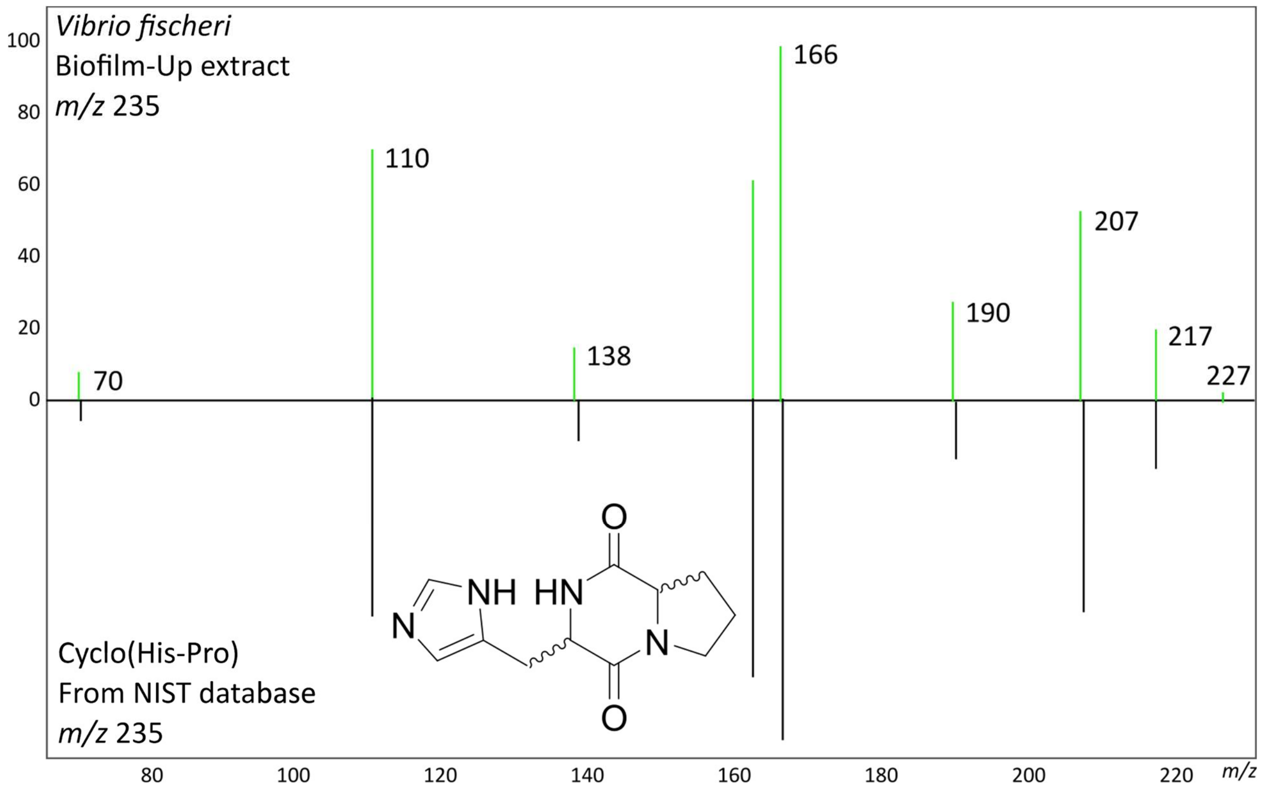

Figure S3. The crude extract of *V. fischeri* Biofilm-Up was queried in the GNPS database and a strong match was calculated between a molecule in the crude extract and cyclo(histidyl-proline), *m/z* 235. The compound was added from the NIST database under the Library Spectrum CCMSLIB00003139663.
