# Supplementary material for: A Small Molecule Coordinates Symbiotic Behaviors in a Host Organ
Source: mBio. 2021 Mar 9;12(2):e03637-20. doi: 10.1128/mBio.03637-20 (PMC8092321; doi:10.1128/mBio.03637-20)
Supplement: FIG S4 [file mBio.03637-20-sf004.pdf]

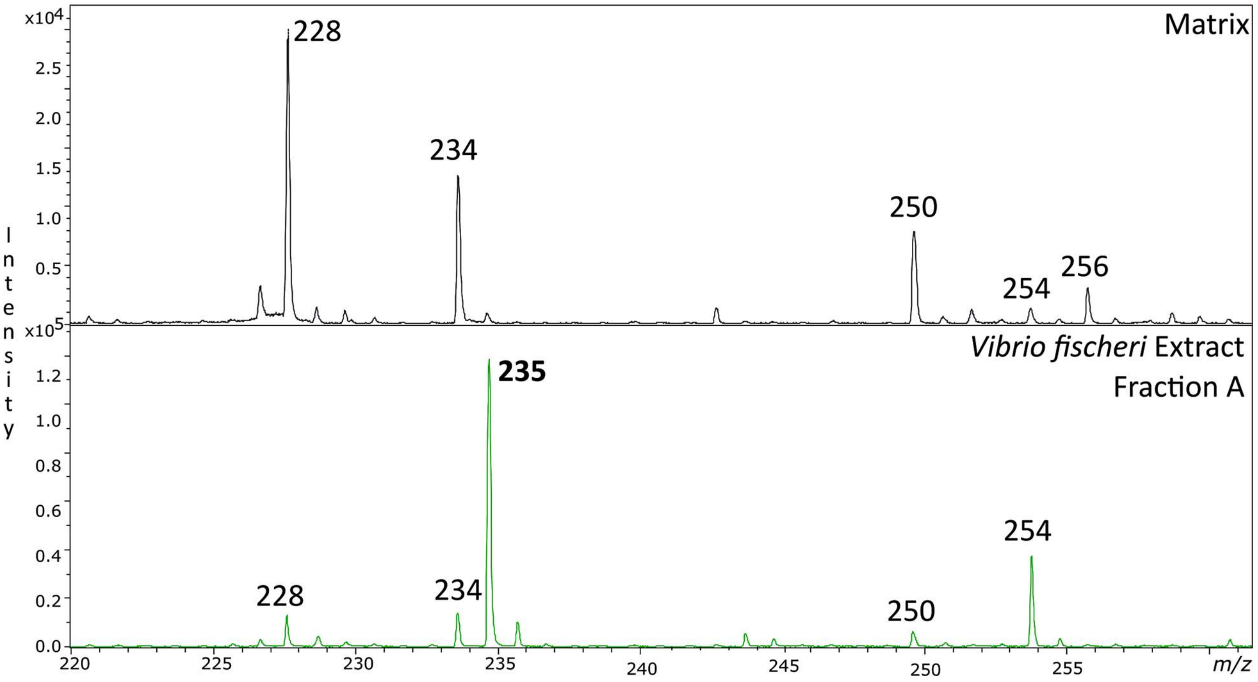

Figure S4. Dried drop analysis of Fraction A from *V. fischeri* Biofilm-Up extract. The *m/z* 235 is present in the extract fraction, but not in the matrix control.
