# Supplementary material for: A Small Molecule Coordinates Symbiotic Behaviors in a Host Organ
Source: mBio. 2021 Mar 9;12(2):e03637-20. doi: 10.1128/mBio.03637-20 (PMC8092321; doi:10.1128/mBio.03637-20)
Supplement: FIG S5 [file mBio.03637-20-sf005.pdf]

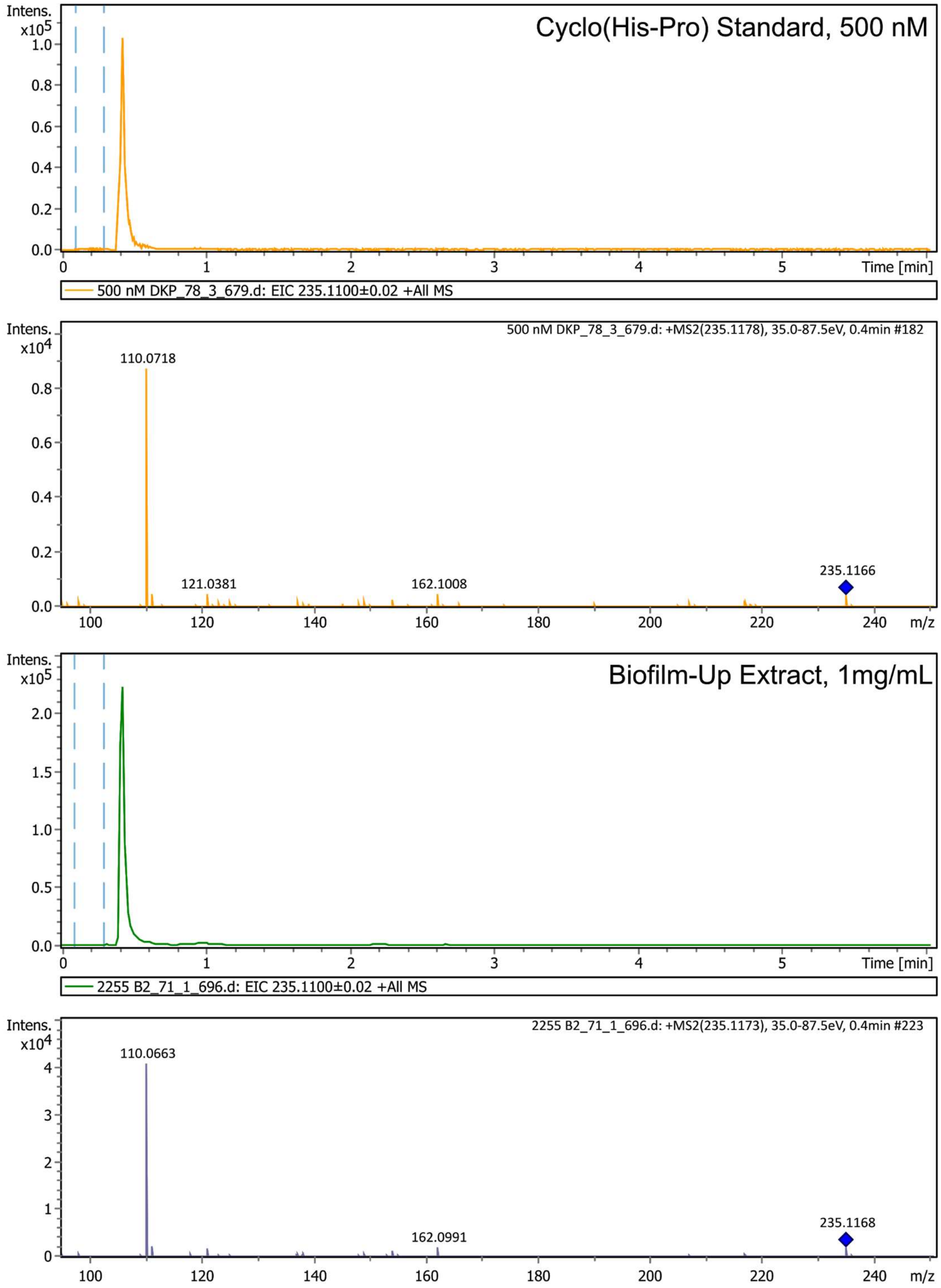

Figure S5. High-resolution electrospray ionization mass spectrometry (HRESIMS) of Cyclo(His-Pro) compared to the extract of Biofilm-Up *Vibrio fischeri* strain. Extracted ion chromatograms (EICs) of *m/z* 235.11 +/- 0.2 Da detected a precursor ion of 235.1178 in the standard, and 235.1173 in the extract (ppm error 7.23 and 6.80, respectively).
