# Supplementary material for: A Small Molecule Coordinates Symbiotic Behaviors in a Host Organ
Source: mBio. 2021 Mar 9;12(2):e03637-20. doi: 10.1128/mBio.03637-20 (PMC8092321; doi:10.1128/mBio.03637-20)
Supplement: FIG S6 [file mBio.03637-20-sf006.pdf]

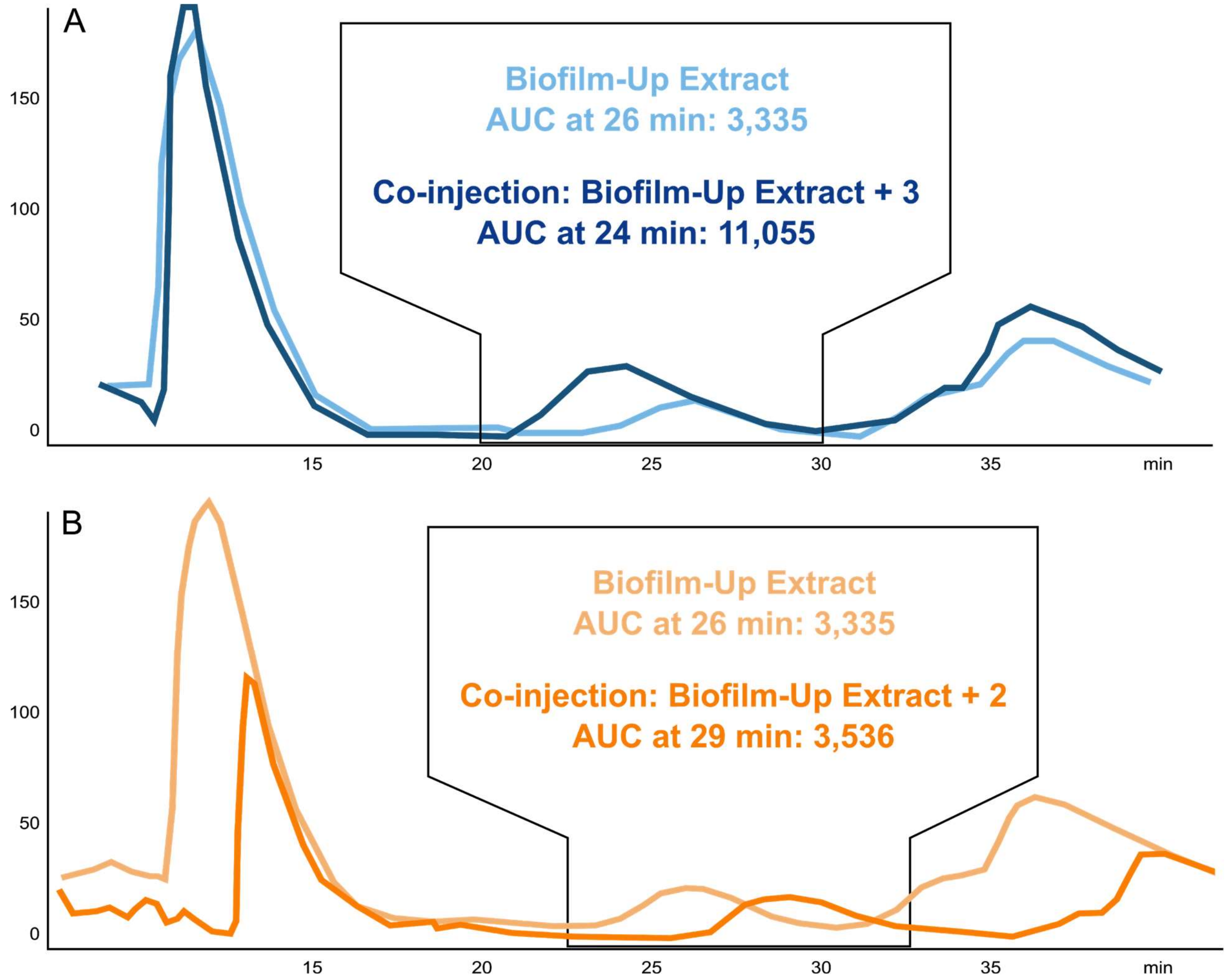

Figure S6. Retention time (RT) matching of stereoisomers compared to Biofilm-Up extract. A peak was observed in the extract at 26 min with an area under the curve (AUC, indicative of the peak intensity) of 3,335. Cyclo(D-His-L-Pro) (**cHP-3**) and cyclo(L-His-D-Pro) (**cHP-2**) eluted from the chiral column at RTs of 24 min and 29 min, respectively, indicating that the peak from the extract at 26 may represent one of the two stereoisomers. **A)** Co-injection of the extract with **cHP-3** resulted in elution of a peak at 24 min whose AUC increased to 11,055. **B)** Co-injection of the extract with **cHP-2** resulted in a peak at 29 min with an AUC of 3,536, indicating that the extract peak and DKP peak did not coalesce, and therefore do not share the same configuration.
