# Supplementary material for: A Small Molecule Coordinates Symbiotic Behaviors in a Host Organ
Source: mBio. 2021 Mar 9;12(2):e03637-20. doi: 10.1128/mBio.03637-20 (PMC8092321; doi:10.1128/mBio.03637-20)
Supplement: FIG S7 [file mBio.03637-20-sf007.pdf]

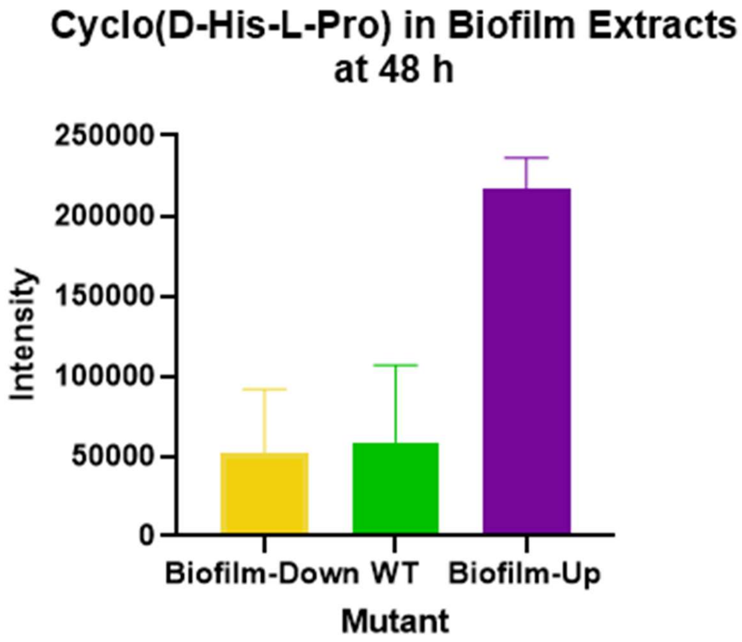

Figure S7. Quantification of **cHP-3** *in vitro* in *V. fischeri* mutants demonstrated that the Biofilm-Up strain produced significantly more of **cHP-3** than the WT and the Biofilm-Down strain.
