# Supplementary material for: A Small Molecule Coordinates Symbiotic Behaviors in a Host Organ
Source: mBio. 2021 Mar 9;12(2):e03637-20. doi: 10.1128/mBio.03637-20 (PMC8092321; doi:10.1128/mBio.03637-20)
Supplement: FIG S8 [file mBio.03637-20-sf008.pdf]

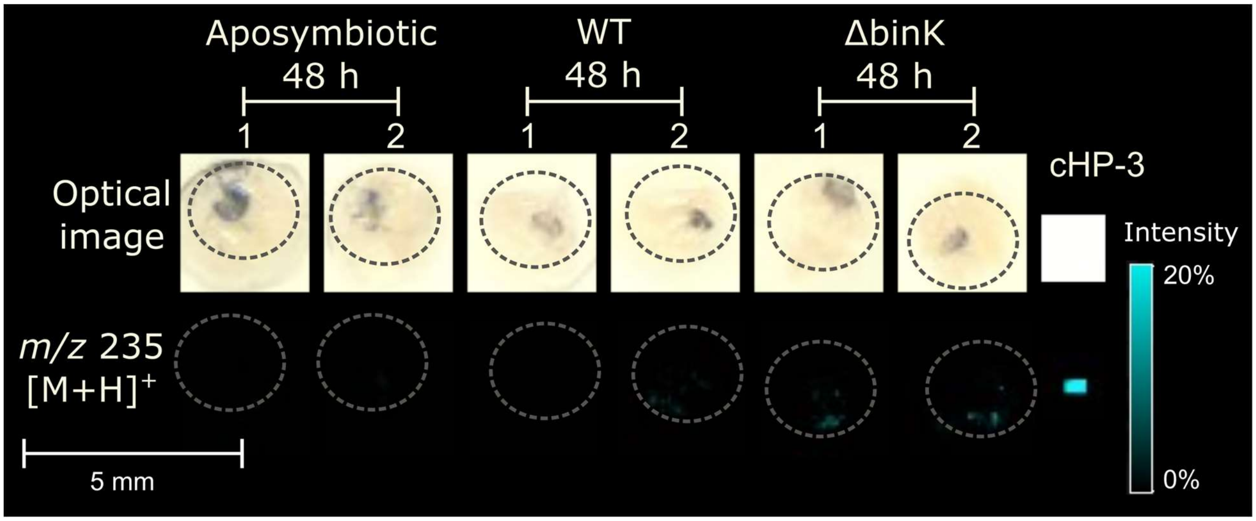

Figure S8. Detection of **cHP-3** in two additional replicates of light organs, with representative images in **Figure 4**. No cHP-3 is detected in light organs with no *V. fischeri* colonized, is detected in the second replicate of a light organ colonized with WT *V. fischeri*, and is detected in two additional replicates of light organs colonized by rscS*binK *V. fischeri*.
