# Supplementary material for: A Small Molecule Coordinates Symbiotic Behaviors in a Host Organ
Source: mBio. 2021 Mar 9;12(2):e03637-20. doi: 10.1128/mBio.03637-20 (PMC8092321; doi:10.1128/mBio.03637-20)
Supplement: FIG S9 [file mBio.03637-20-sf009.pdf]

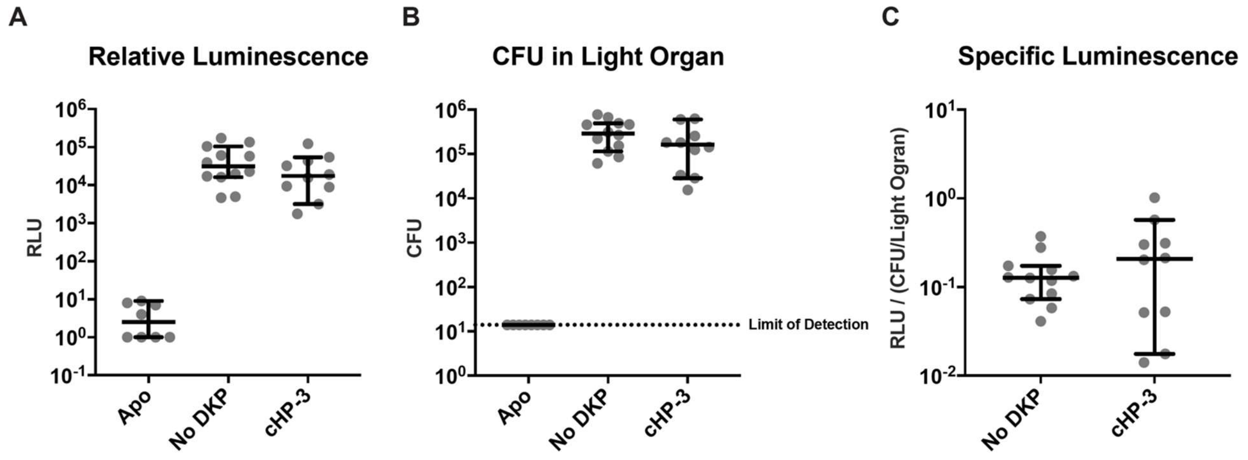

Figure S9. Colonization of squid hatchlings with exogenous cHP-3. Aposymbiotic hatchling squid were inoculated for 3 h with strain ES114 and then maintained for 48 h. Squid were kept in various conditions throughout the experiment: "Apo" which is the aposymbiotic condition where no bacteria were inoculated, "No DKP" where squid were inoculated but no exogenous molecule was added, and "**cHP-3**" where 250 μM of **cHP-3** was present in the water. A) The bioluminescence of each individual squid was measured and normalized to a blank reading. Units are relative luminescence units. B) Bacterial load in the light organs of individual squid was determined by counting colony forming units (CFUs) of plated dilutions of individual homogenized squid. C) The specific luminescence, or amount of luminescence from each bacterial cell, was calculated by dividing the relative luminescence by the total CFU in the light organ. For all graphs, bars indicate the median with a 95% confidence interval. Graphs represent a pool of three biological replicates for a total of n=7-12 for each condition.
